# Dispensing antiretrovirals during Covid-19 lockdown: re-discovering community-based ART delivery models in Uganda

**DOI:** 10.1186/s12913-021-06607-w

**Published:** 2021-07-13

**Authors:** Henry Zakumumpa, Christopher Tumwine, Kiconco Milliam, Neil Spicer

**Affiliations:** 1 Makerere University, School of Public Health, Kampala, Uganda; 2 Kabale University, School of Medicine, Kabale, Uganda; 3 Department of Sociology, Kyambogo University, Kampala, Uganda; 4 London School of Hygiene and Tropical Medicine, London, UK

## Abstract

**Background:**

The notion of health-system resilience has received little empirical attention in the current literature on the Covid-19 response. We set out to explore health-system resilience at the sub-national level in Uganda with regard to strategies for dispensing antiretrovirals during Covid-19 lockdown.

**Methods:**

We conducted a qualitative case-study of eight districts purposively selected from Eastern and Western Uganda. Between June and September 2020, we conducted qualitative interviews with district health team leaders (*n* = 9), ART clinic managers (*n* = 36), representatives of PEPFAR implementing organizations (*n* = 6).In addition, six focus group discussions were held with recipients of HIV care (48 participants). Qualitative data were analyzed using thematic approach.

**Results:**

Five broad strategies for distributing antiretrovirals during ‘lockdown’ emerged in our analysis: accelerating home-based delivery of antiretrovirals,; extending multi-month dispensing from three to six months for stable patients; leveraging the Community Drug Distribution Points (CDDPs) model for ART refill pick-ups at outreach sites in the community; increasing reliance on health information systems, including geospatial technologies, to support ART refill distribution in unmapped rural settings. District health teams reported leveraging Covid-19 outbreak response funding to deliver ART refills to homesteads in rural communities.

**Conclusion:**

While Covid-19 ‘lockdown’ restrictions undoubtedly impeded access to facility-based HIV services, they revived interest by providers and demand by patients for community-based ART delivery models in case-study districts in Uganda.

## Background

The Covid-19 pandemic has had important impacts on access to health services globally, but particularly so in Sub-Saharan Africa which has an overwhelming infectious diseases burden [1–3].

As part of Covid-19 prevention measures, several countries in Sub-Saharan Africa implemented stringent ‘lockdown’ measures. These included bans on public transport and the prohibition of mass gatherings that are conducive for infection spread [4, 5]. In addition, standard prevention measures such as wearing face masks, social distancing and hand sanitizing have become the ‘new normal’ in many sub-Saharan countries [4, 5].

‘Lockdown’ measures were recommended by the World Health Organization (WHO) and were informed by epidemic control experiences from China, Western Europe and North America [6, 7]. There is some evidence that ‘lockdown’ measures have contributed to reducing Covid-19 infection rates [6, 7].

While the public health imperative of ‘lockdown’ measures is largely clear, its impact on access to general health services, such as maternal and newborn care and HIV care and treatment is beginning to become apparent [8, 9].

In Uganda, ‘lockdown’ measures were implemented from March 2020 [10]. The Ugandan government announced a ban on public and private transport, the closure of all educational institutions and public entertainment facilities and the enforcement of a national curfew [10]. Due to restricted movements, special permission was required for private individuals to travel. Uganda has a decentralized health system whereby sub-national units known as districts retain overall responsibility for social service provision [11, 12]. In this context, permission to travel was to be sought from designated public officers known as ‘Resident District Commissioners’ [11].

‘Lockdown’ measures in Uganda had an immediate impact on HIV services particularly on access to ART refills for the over 1.2 million Ugandans receiving antiretroviral therapy (ART) [13, 14]. Recipients of HIV care could no longer make in-person visits to facilities for scheduled reviews or for accessing their medication refills. Furthermore, ‘lockdown’ impeded the ongoing implementation of differentiated ART delivery models [14]. Since 2017, the Ministry of Health of Uganda has been implementing five differentiated ART delivery models. These include community-based ART delivery through patient-led ART refills delivery (Community Client-Led ART delivery or CCLAD) and Community Drug Distribution Points (CDDPs) [14, 15]. Less-intensive facility based ART delivery models include Fast Track Drug Refills (FTDR), which entail a three to six-month supply of ART medicines (freed from clinical reviews) on visits by patients to facilities [14, 15]. Indeed, multi-month ART dispensing is seen as a cornerstone of differentiated ART delivery in Uganda and in other countries with a high HIV burden [16–19].

The ban on public transport and private means of travel in Uganda effectively impeded facility-based HIV care as patients were severely constrained in physically accessing points-of-care.

While there has been a steadily emerging evidence base on the effects of ‘lockdown’ measures on access to health services in general [1–3], there is little research on the notion of health-system resilience with respect to differentiated ART delivery in the face of the Covid-19 pandemic [20]. Health system resilience has been defined as ‘*the capacity of health actors, institutions, and populations to prepare for and effectively respond to crises; maintain core functions when a crisis hits; and, informed by lessons learned during the crisis, re-organize if conditions require it’* [21]. Examples of the notion of health system resilience include strategies by health workers for reaching patients in their households and communities owing to statutory travel restrictions and innovations around distribution of medications in the context of bans on public transport. The notion of health system resilience has also been studied with respect to the Ebola outbreak in Western Africa [21].

Given that Sub-Saharan Africa is lagging behind in global efforts to roll out the Covid-19 vaccine, the effects of the pandemic are likely to last longer there [22]. Hence, strategies for mitigating the impact of Covid-19 prevention measures on access to HIV care and treatment services is critical [20, 23]. Documenting innovations around dispensing antiretrovirals in resource-limited settings is beneficial to frontline providers, recipients of HIV care, national-level HIV programme managers and major HIV donors such as PEPFAR [23]. Furthermore, the Covid-19 pandemic may represent new opportunities for innovation in health services delivery and re-imagining health-systems in general [20, 23].

This study starts to fill this knowledge gap. The paper explores health system resilience at the sub-national level in Uganda with regard to strategies for dispensing antiretrovirals during Covid-19 lockdown restrictions.

## Methods

### Research design

We adopted a qualitative case-study design. We utilized a qualitative approach because we aimed to explore the notion of health-system resilience [21] from the perspective of diverse actors in district health systems [24, 25] namely frontline health workers, district health teams and recipients of HIV care.

### Analytical framework

The study design was conceptually informed by an analytical framework advanced by Lévesque and colleagues [26] which emphasizes a multi-level analytical lens in understanding the complex dynamics involved in access to healthcare which incorporates the health-system, organizations, providers, individual-level and contextual factors. The framework informs our selection of a diverse set of participants ranging from representatives of international donors, district-level actors, facility-level personnel and patients as clients of the health system [27].

### Study sites and sampling

We chose the district as a study unit because in Uganda’s decentralized health-system set up, the district is the sub-national administrative unit that retains overall responsibility for provision of social services [28, 25].We purposively selected eight districts in Eastern Uganda (Mbale, Manafwa, Bududa, Sironko, Bulambuli) and Western Uganda (Kabarole, Kyegegwa, Kyenjonjo). These sub-regions have a relatively high HIV burden in Uganda [29]. Furthermore, we sought to explore differences in geographical contexts between the two regions. For instance, Eastern Uganda (especially the Elgon region) is known to be mountainous which potentially impacts access to health services [27].

We aimed to achieve diversity in our study sample by; a) level of care in the Ugandan health system (tertiary/secondary/ primary level facilities) [30], b) facility ownership-type (public/private) and c) setting (urban/rural). The characteristics of participating facilities are represented in Table 1**.**

**Table 1:** Characteristics of participating facilities

–	–	Setting	
Level of service delivery	N	Urban	Rural
Regional Referral Hospital	2	1	1
General Hospital	6	3	3
Health centre IV	10	6	4
Health centre III	6	2	4
Total	24	12	12

### Data collection

#### Qualitative interviews

Qualitative data were collected between June and September 2020. We conducted eight face-to-face key informant interviews (KIIs) with at least one District Health Officer in each case-study district. In-depth Interviews (IDIs) were conducted with 16 ART clinic managers (five doctors, eight clinical officers, three nurses), on-site, in their offices, at participating facilities to understand provider-level strategies for overcoming ‘lockdown’ measures for distribution of ART refills. Six in-depth interviews were conducted with representatives of regionally-based PEPFAR implementing organizations to explore the role of HIV donors in Uganda in mitigating the impacts of ‘lockdown’. All interviews were conducted in English by the first author who has an academic background in the social sciences and extensive experience in qualitative research in HIV services. The first author was assisted by three research assistants who took notes during the proceedings and operated the audio recorder. Investigators complied with Uganda National Council for Science and Technology (UNCST) 2020 guidelines on conducting research in the context of the Covid-19 pandemic. To this end, we observed Ministry of Health Standard Operating Procedures (SOPs) for prevention of Covid-19 infection such as implementing social distancing requirements, the use of facemasks and the use of alcohol-based hand sanitizer.

#### Focus group discussions

To gain an in-depth understanding of the experiences of patients during ‘lockdown’ as a group [31], we conducted six focus group discussions (48 participants) with patients receiving HIV care at case-study facilities (Table 1**).** A topic guide was constructed prior to the conduct of FGDs and was framed around factors influencing healthcare based on the adopted analytical framework [27]. Examples of notions derived from the framework which were probed in the focus groups include a) *Ability to reach health care* which ‘relates to the notion of personal mobility and availability of transportation’ to enable physical access to health centres [27] b) *Affordability*: this denotes ‘the economic capacity for people to spend resources and time to use appropriate services'. It results from direct prices of services and related expenses in addition to opportunity costs related to loss of income’ [27].

The focus groups were gender-disaggregated. Three focus groups were held with adult males and three were conducted with adult females. The patients who participated in the FGDs were selected with the help of ART clinic managers at participating facilities based on the study objectives which was described by investigators. The demographic characteristics of participants in our focus groups is shown in Table 2.

**Table 2:** Characteristics of participants in focus groups

CHARACTERISTIC	Frequency (***n = 48***)	Percent (100%)
**Gender**		
Male	20	41.6
Female	28	58.3
**Age range**		
18–24	6	12.5
25–34	6	12.5
35–44	12	33.3
45–54	18	37.5
55–64	6	12.5
**Marital status**		
Married	14	29.1
Single	28	58.3
Widowed	6	12.5
**Mode of travel to HIV clinic**		
Bicycle	12	25.0
Public transport	20	41.6
Walk	16	33.3

The category of all participants we engaged in this study is shown in Table 3**.**

**Table 3:** Category of participants (*n* = 99)

Respondent type	Number
District Health Team leaders	09
ART clinic managers	36
Representatives of regionally-based PEPFAR Implementing Partners (IPs)	06
**Focus Group Discussions**	06
Recipients of HIV care	48

### Data analysis

Interviews and focus groups were audio-recorded and transcribed verbatim by four research assistants. The transcripts were subsequently uploaded into Atlas.ti for data management and analysis.

We followed the procedures recommended for qualitative data analysis by Miles and Huberman (1994) [32]. To this end, data were analyzed in an iterative process involving four major stages [19, 33]. The first step involved *data familiarization* through multiple readings of interview transcripts by HZ, NB, MB [32]. The second step entailed *generating a coding framework* (using ATLAS. ti). Codes were inductively generated from the interview transcripts in a team-based process involving four authors (HZ, NB, MB, AB) [34]. The third stage was that of *abstracting the coded data into thematic categories.* The emergent themes were inductively-derived. The fourth and final step was that of *Overall interpretation and synthesis* [17]*.*

## Results

The results presented below, based on our qualitative interviews, reflect the five broad strategies that emerged in our analysis of provider Covid-19 ‘lockdown’ mitigation responses (Table 4). The five strategies identified were: a) intensifying home-based ART refill deliveries; b) extending multi-month ART dispensing from three to six months; c) piggy backing off Covid-19 response outreach in the community for medication distribution; d) leveraging the Community Drug Distribution Points (CDDPs) model; and f) increased reliance on health information systems to support ART refills distribution.

**Table 4:** Emergent themes and sub-themes

Theme	Sub-themes
Home-based ART deliveries	• Dedicated vehicle fleets for home-based ART refills delivery. • Expert patients as members of ‘mobile brigades’
Leveraging CDDPs	• Shifting ART refills distribution to outreach sites in the community. • One peer-leader- per-sub-county refill distribution model.
Scaling up multi-month dispensing (MMDs)	• Extending refills from 3 to 6 months. • ART refills to ‘visitor’ patients. • Longer-term orders for ART supplies.
Increased reliance on health information systems	• Use of patient data bases for locating physical locations. • Use of geo-spatial technologies
Leveraging Covid-19 response funding	• Utilizing Covid-19 community outreaches to deliver ART refills. • Leveraging ‘Covid-19’ funding for fuel for ART refills distribution.

### Intensifying home-based ART refill delivery

To mitigate the impact of Covid-19 ‘lockdown’ on physical access to facility-based HIV care, providers aggressively intensified home-based deliveries of ART refills to patients. Due to a Uganda government ban on public and private transport, patients could no longer travel to facilities to access ART refills based on previously determined schedules. One public regional referral hospital, and nongovernmental providers such as TASO (The AIDS Support Organization), reported assigning dedicated vehicle fleets to distributing ART refills to the known physical addresses of patients within rural communities. Select ‘expert patients’ constituted part of these ‘mobile brigades’ that traversed rural communities and helped in identifying patients’ homes to dispatch medication packages. The regionally-based PEPFAR implementing organization in Eastern Uganda availed part of the needed vehicles and fuel to enable health facilities traverse communities delivering ART refills.

*‘We supported health facilities to do home- to- home drug delivery. So we took the drugs directly to their (patients) homes. For some we were able to reach their homes. Those whose homes we couldn’t locate, we would make an arrangement to deliver the drugs to a common place in their locality where they would gather and pick their drugs’ [Representative, PEPFAR implementing organization, Eastern Uganda]*

Some health workers reported taking personal initiative in extending ART refills to patients who lived in their neighbor hoods or those within a five-kilometer radius.

### Piggybacking off Covid-19 response activities for ART distribution

At the level of district health teams, two District Health Officers (DHOs) reported that they piggybacked off Covid-19 response transport funding provided to districts for tracing ‘alerts’ in the community to also distribute ART refills in the households of recipients of HIV care.

*‘I used the opportunity of having authority over several vehicles which were at my disposal as part of the Covid-19 outbreak response at the district. They sent me about 65 million ($ 17,808) of which about 40% was for fuel. So the only way I could help was to deliver ART refills through our Covid-19 epidemiological response in a kind of outreach model. [District Health Team leader, Eastern Uganda].*

The DHOs further indicated that they made some buses available for ferrying health workers to facilities. Health workers were equally affected by the government ban on public transport. The handful of patients who were able to physically access facilities reported being pleasantly surprised at the early reporting times of health workers who benefited from this provision of free transport.

*‘Covid-19 came with some positives. Health workers arrived much earlier than usual because they had buses ferrying them. We kept getting calls from patients and community at large appreciating the fact that health workers were arriving early at work and staying much longer at facilities because they were assured of transport back home’ [District Health Team leader, Western Uganda].*

However, home-based ART refill distribution was not without constraints. HIV-related stigma was frequently cited by participants as a major constraint. This was manifested in two forms. It emerged that the physical addresses registered by patients at the facilities frequently turned out to be incorrect due to the fear by patients of unintentional disclosure of their HIV status. Secondly, in the case of TASO, a renowned HIV care provider in Eastern Uganda, respondents mentioned that the vehicle fleet used in ART refills distribution was branded with the TASO logo and brand colours. Whenever a TASO vehicle was sighted at a household, it was almost certain that the household had a person living with HIV. Hence, community HIV-related stigma impeded the full potential of the home-based ART refills delivery in reaching multitudes of patients within communities.

*‘The challenge encountered with home-based deliveries was that of stigma. You are talking to a patient on phone trying to locate exactly where they are after arriving in their neighborhood. He is saying ‘I am nearby. I am around’. But because there are many people in the vicinity he fears to be seen approaching a TASO-branded Land Cruiser (vehicle). Many times we would go back with their medication packages and leave in frustration’ [Patient peer-leader, nongovernmental facility, Western Uganda].*

### Leveraging the community drug distribution points (CDDPs) model

Prior to the Covid-19 ‘lockdown’, the CDDPs delivery model (where outreach sites within communities are designated for ART refill pick-ups) had registered the lowest uptake in all of the five differentiated ART delivery models endorsed by Uganda’s Ministry of Health [35]. However, with Covid-19 ‘lockdown’, facility-based HIV care was severely impeded. Given this context, the CDDPs model gained an increased importance in case-study districts. TASO, a leading nongovernmental ART provider in Uganda, reported shifting the bulk of its ART refills distribution to outreach sites (CDDPs) deep within the community to reach patients held up by lockdown measures. *Boda boda (motor cycle taxi),* a dominant form of transport in rural Uganda, was restricted during lockdown. This rendered CDDPs a critical outlet for refill pick-ups. Patients frequently use these taxis as transport and therefore their absence renders CDDP as an attractive option.

#### Involvement of patient peer-leaders in ART refills distribution

Participants from TASO reported that they intensified counselling of patients by telephone to enhance their willingness to pick up their ART refills from Community Drug Distribution Points (CDDPs). Prior to the Covid-19 ‘lockdown’, HIV-related stigma was said to be a fundamental barrier to patient enrollment in community-based ART delivery models.

TASO counselled their patients who were still receiving facility-based care to overcome their internalized stigma and encouraged them to collect their refills at CDDP points in remote outreach sites.

*‘For us we went the extent of encouraging patients to join CDDPs or receiving their drugs from the community. The Covid-19 crisis helped us so much in getting patients to accept to join CDDPs. We intensified health talks especially during Covid-19 ‘lockdown’ to encourage patients who hadn’t yet joined, to join CDDPs near where they live’ [Patient peer-leader, private not-for-profit, Eastern Uganda]*

It emerged from interviewees’ comments that the Covid-19 ‘lockdown’ saw a marked increase in uptake of community models due to patients’ inability to physically access facilities owing to movement restrictions. Health workers indicated that in their Covid-19 ‘lockdown’ experience, sustained counselling of patients contributed to a significant increase in uptake of community-based ART delivery including those who had initially indicated a preference for facility-based care.

At two regional referral hospitals, health workers indicated that they aggressively scaled-up ART refill distribution through leaders of patient groups in the Community Client-Led ART Drug delivery model (CCLAD). CCLADs are voluntary groups comprising of up to six patients living in the same neighborhood who rotate in picking up ART refills from facilities on behalf of each other. Health facilities leveraged the CCLAD model during the ‘lockdown’ to reach a multitude of patients through their group leaders including reaching patients who reside in hard-to-reach areas in the mountainous Elgon sub-region in Eastern Uganda. In one of the case-study districts in Eastern Uganda, a district health officer (DHO) utilized a strategy of delivering ART refills through the use of *boda boda* in a predominantly rural setting. Instead of ART refill delivery to individual patient leaders of CCLAD groups, one peer-leader was selected for each of the 16 sub-counties that make up the district.

The sub-county group leader would in turn physically pass on the medication packages to individual patient leaders within that sub-county who would then reach individual patients in an innovative supply chain network.

*‘We selected an overall peer-leader to deliver ART refills to patients we could locate in each of the 16 sub-counties in our district. I was the one selected for my sub-country and I contacted each of the leaders of the patient groups in my area and delivered medication packages for patients under their voluntary group’ [Patient peer-leader, Regional Referral Hospital, Eastern Uganda].*

### Extending multi-month dispensing from three to six months

Whenever supply chains permitted**,** providers reported that ART refills were extended from three to six months for patients deemed clinically stable on ART. Prior to Covid-19, the Uganda Ministry of Health was recommending a three-month medication supply for patients deemed clinically stable on ART. However, due to the ban on travel during Covid-19 lockdown, patients were scarcely able to make in-person visits to the facilities. Extending multi-month ART dispensing from three to six months came as a huge relief to patients.

*‘At TASO we have been doing MMD (multi-month dispensing). For stable clients we have been giving them a six-month supply of ART while for unstable clients we have been giving them a three-month supply and that is still going on. I was told that we have sufficient (ART) stock to last an additional three months ahead’ [ART clinic manager, non-governmental provider, Eastern Uganda].*

Even when the ban on public transport was partially lifted by the Uganda government in June 2020, many patients could no longer afford public transport due to a hike in prices of public transport occasioned by social distancing requirements in public transport commuter vans. Because transporters ferried less passengers due to social distancing requirements, their revenues contracted, leading to an increase in the per-person fare. Hence, the effects of the Covid-19 prevention measures lingered on even after the most stringent measures were lifted by the Uganda government. Patients reported an increasing difficulty in affording public transport to visit facilities for ART refill pick-ups due to the loss of wage income from small and medium enterprises (SME) businesses many of which were negatively impacted by Covid-19 ‘lockdown’. Patients reported that they were scarcely able to buy food due to ‘hand-to-mouth’ livelihoods adversely impacted by ‘lockdown’ measures.

*‘The challenge we had with Covid-19 is that our people work hand-to-mouth. There is a challenge of people being able to afford food. Many patients can’t swallow drugs because they have no food because they were not able to work to put food on the table’ [Patient, sub-district health facility, Western Uganda].*

It is important to note that whereas extending multi-month dispensing was timely due to the obtaining circumstances, patients reported that it could have contributed to stock-outs at some facilities which did not have the capacity to implement them due to limitations in supply chain capacity. Health workers explained that stock-outs occurred because Covid-19 ‘lockdown’ impeded ART supply chains. Hence, whereas providers endeavored to provide longer ART refills to patients who they could physically access, it depleted the available stock for the rest of patients.

#### The impact of ‘visitor’ patients on ART stock availability

Our interviews with health workers in Western Uganda brought to light the ‘visitor’ phenomenon’s impact on the available ART stocks during ‘lockdown’. A District Health Officer (DHO) in the Rwenzori sub-region in Western Uganda reported that the Ministry of Health in its Covid-19 mitigation guidance allowed health facilities in Uganda to provide ART refills to ‘visitor’ patients or those who ordinarily attend care at other facilities.

*‘One of the things we have seen is that we have had an influx of ‘visitor’ patients coming to pick medicines during the ‘lockdown’. So we found that we had not planned for them to come and pick medicines. So we found that what we had planned to give to our regular patients was less. Hence, for those who were to receive a two-month supply ended up getting a one-month supply and others ended up with medicines to last only two weeks’ [District Health Team, Western Uganda].*

Due to HIV-related stigma, patients frequently bypass the nearest ART sites to their homes. Indeed, it is not uncommon for patients to seek HIV care hundreds of kilometers away from their homes. However, Covid-19 ‘lockdown’ restrictions compelled them to seek care at ART sites closest to their homes. Health facilities were provided with telephone contacts of ART-providing facilities in their sub-region to allow providers access patient information such as the ART regimens they were on so that they could provide the correct medication to ‘visitor’ patients.

*‘The innovation of sharing telephone contacts of health workers from different health facilities in neighboring districts where these patients are coming from was a very good initiative because we would be able to call the other end and if the patient did not have their records with them we would ascertain on what regimen they were on and how they were taking the medicines. If he or she was suppressed or not, or if they had any new problem with them. We even got to know their medical history, so that was very good to reduce any challenge. [District Health Team, Western Uganda].*

#### Longer-term orders of ART commodities

Private sector providers such as TASO reported that they made longer-term ART commodities supply orders with their main supplier Joint Medical Stores (JMS) which is a leading supplier of HIV commodities to the private health sector in Uganda. From the perspective of providers, it emerged that multi-month dispensing demanded unprecedented ART commodity stocks owing to the aggressive ART refills distribution through intensified community-based delivery platforms. Public facilities in Uganda are supplied ART commodities based on a bi-monthly order cycle which could not meet the performance demands of a six-month supply of antiretrovirals as a ‘lockdown’ mitigation strategy. However, nongovernmental providers such as TASO were more flexible in their supply chain strategies and utilized this decision space to place longer-term orders with private commodity suppliers.

*‘We placed another order with JMS (Joint Medical Stores) to enable us have a sufficient stock of drugs to take us up to March 2021 (nine months ahead)’ [ART clinic manager, private not-for-profit, Eastern Uganda]*

### Increased reliance on information system technologies

Health workers reported that Covid-19 ‘lockdown’ compelled them to utilize health information systems in an unprecedented way in order to reduce their burgeoning cases of lost- to- follow-up. Health workers relied on health information systems in order to reach patients trapped in their homesteads during ‘lockdown’.

#### Use of geospatial technologies

A public regional referral hospital (RRH) participating in this study reported that they utilized geospatial modeling to locate the physical addresses of the homesteads of recipients of HIV care in their predominantly rural settings that are not adequately mapped with modern physical addresses. Using the available information about patients within their data bases such as phone numbers and physical addresses they attempted to locate geographical points where patient reside and subsequently linked them with their ‘mobile brigades’. Motor cycles were frequently used to deliver pre-packaged medication to identified physical addresses deep inside rural communities. District health teams in Eastern Uganda availed a fleet of motor cycles and a dedicated fuel fund made available to health facilities for this purpose. The PEPFAR implementing organization based in in this sub-region also contributed a vehicle fleet to aid in transporting medication packages to individual addresses across the four districts in their purview.

#### Use of telephone hotlines for ART refills distribution

Three tertiary-level hospitals reported that they set up telephone hotlines for extending ART refills to patients. Telephone hotlines were used to enable patients reach the hospitals and help pin point geographical locations where ART refills could be delivered. A regional referral hospital indicated that they set up four ‘land line’ telephone hotlines that were manned by ‘expert patients’ who engaged in constant communication with patients held up by the ‘lockdown’.

*‘We have a hotline where patients who are able to, call using our landline. So, whoever would call we would go to their homes within the community and do home-based drug delivery. So, we took the drugs to their homes. For patients whose homes we couldn’t geographically locate, we would make an arrangement for them to receive their ART refills at a nearby place in their locality which they could easily identify. Then the patients would assemble there and receive their refills’ [Expert patient, public facility, Western Uganda].*

One of the barriers encountered in running the hotline for Ugandan hospitals based near the international border with Kenya was that several Ugandan patients who earn livelihoods in neighboring Kenya could not access their ART refills in Uganda across the common border on account of closure of the international border between the two countries during Covid-19 ‘lockdown’.

## Discussion

Although there is a steadily emerging evidence base on the impact of the COVID-19 pandemic on access to health services, the related notion of health-system resilience with respect to ART refill distribution has received little empirical attention. Utilizing a case-study of eight districts in Uganda, we sought to understand strategies adopted at the sub-national level [25] for dispending antiretrovirals in the context of a ban on public and private transport as part of ‘lockdown’ measures. Five broad strategies for ART distribution emerged in our study. We found that home-based delivery of ART was aggressively scaled-up. Providers leveraged the Community Drug Distribution Points (CDDPs) model and re-routed the bulk of ART distribution from facilities to outreach sites within their predominantly rural community catchment areas. Multi-month dispensing for stable patients was extended from three to six months. There was an increased reliance on health information technologies to locate and reach patients in the largely unmapped rural-based homesteads by use of geospatial modeling and the use of patient data bases to help pin point geographical locations of households. Districts piggy backed off Covid-19 response community outreaches to distribute antiretrovirals.

In this study, we found that home-based ART delivery was a widely implemented strategy for reaching patients held up by ‘lockdown’ measures across the eight case-study districts. The Covid-19 pandemic experience in Uganda reinvigorated interest in home-based ART delivery as a strategy for ART distribution that is worthy of further research. Although home-based ART delivery is not one of the five differentiated ART delivery models endorsed by the Uganda Ministry of Health [15], and the evidence is mixed on its cost-effectiveness [36], the largely successful emergency implementation of this model merits further consideration. In our study, patients expressed satisfaction with deliveries of ART refills to their homes. Previous studies have reported the non-inferiority of home-based HIV care [37], including one conducted in Uganda [31]. In South Africa, Mkumbang and colleagues [32] posit that home delivery of pre-packaged ART medication was a preferred model of delivery in the context of Covid-19 containment measures there. It is important to point out that HIV-related stigma stood out as a fundamental impediment to home-based ART delivery but also generally in strategies around decentralization of ART refill distribution which calls for appropriate interventions [38]. Previous studies have reported HIV-related stigma as a fundamental barrier to differentiated ART delivery [15, 17, 19]. Future research would do well to reassess the cost effectiveness of home-based ART delivery as an additional option among the array of differentiated ART delivery models endorsed by the Uganda Ministry of Health.

In this study we found that providers leveraged Community Drug distribution points (CDDPs) for ART refills distribution. A non-governmental provider reported that they had routed the bulk of their ART refills distribution through CDDPs during the ‘lockdown’ phase. Although previous studies have reported a relatively low uptake of community drug distribution points in Uganda [35, 39], in this study, it emerged that CDDPs gained an increased importance in the context of restricted movements during ‘lockdown’ in Uganda. Furthermore, providers reported that through telephone counseling, patient uptake of community based ART increased markedly compared to the pre- ‘lockdown’ phase. Participants from TASO, one of the leading ART providers in Uganda, reported that sustained counseling of patients and community engagement enhances uptake of community models. Although several studies report patient preferences for facility-based HIV care [18, 19, 39], our study findings suggest that sustained community engagement can help patients overcome psycho-social barriers to enrollment in community-based models of HIV care [40–43]

Across our eight case-study districts from Western and Eastern Uganda, it emerged that providers extended ART refills from the approved three-month supply to six months for clinically stable patients. Although previous studies have reported the implementation of six-month ART dispensing, in countries such as Zambia [44], Malawi [45] and Zimbabwe [46], in Uganda this had not been implemented at routine points-of-care prior to Covid-19 lockdown. Our study findings suggest that six-month dispensing is feasible in Uganda from the perspective of providers. However, supply chain capacities need to be strengthened in Uganda to enable implementation at the facility-level to reduce stock-out events [41, 47]. This may require national-level process re-engineering of supply chains such as bolstering storage capacities [39], restructuring from a bi-monthly order cycle to an architecture that supports multi month dispensing [48]. In this study we found that providers who implemented six month dispensing without strengthened supply chain capacity, inadvertently contributed to stock-out events that negatively impacted the stock of ART commodities available to the broader base of patients.

 We found that facilities increasingly relied on information technologies and patient data bases to locate geographical locations of homesteads to enable home-based delivery of ART refills. The use of geospatial modeling technologies for pin pointing locations of households of recipients of HIV care stood out. Our findings suggest that use of geographical information systems in ART distribution could represent a new frontier in health services delivery in rural Sub-Saharan Africa. Rural settings dominate in Uganda and the broader Sub-Saharan Africa region (more than 80%) [49]. Given this backdrop, formal physical address coverage is very low indeed. Hence, using innovative geographic technologies to locate patient residences in rural settings in Uganda could enhance the uptake of differentiated anti-retroviral therapy services (DARTS) [17–19, 39, 44–46] and health services in general. DARTS emphasizes reducing unnecessary burdens on health systems especially with regard to stable patients who can access care in out-of-facility platforms [17–19, 39, 44–46]. Several studies have documented the importance of geospatial technologies for improving HIV service coverage in Sub-Saharan Africa [49–51].

In this study we found that patients expressed increasing inability to afford public transport to facilities for drug pick-ups owing to hikes in fares due to social distancing provisions implemented in commuter vans in Uganda. In addition, several patients reported not being able to afford routine meals or simply-buying food during Covid-19 ‘lockdown’ and its immediate aftermath. This raises concern around the effects of Covid-19 prevention measures on ART adherence at the individual-level and impacts on viral suppression at the population-level [52]. This calls for further research to understand the impacts of Covid-19 on viral suppression in Ugandan patients especially those utilizing longitudinal data with a retrospective lens [48, 53].

### Limitations

We sought to explore the notion of health system resilience from the perspective of sub-national actors in eight purposively selected districts from Eastern and Western Uganda [25]. As such, our study sample was not nationally-representative. Utilizing a case-study approach has inherent limitations in the generalizability of study findings [24]. In addition, the patients who participated in our focus groups were more representative of those who were able to overcome transport barriers and come to collect their medications at case-study facilities.

However, our study has several strengths. We paint an in-depth picture of decision space by actors at the sub-national level in Uganda in mitigating the impacts of Covid-19 ‘lockdown’ in order to extend ART refills to patients caught up in their homes in rural Uganda. In addition, we adopt a multi-level analysis lens capturing a diverse set of stakeholders that incorporates district health teams, regionally-based donor ‘implementing organizations’, facility-level workforce and patient-level perspectives. Beyond documenting strategies for ART distribution during ‘lockdown’, we also capture the impact of Covid-19 ‘lockdown’ on patients within Uganda’s decentralized settings.

## Conclusion

While Covid-19 ‘lockdown’ restrictions undoubtedly impeded access to facility-based HIV services, they revived interest by providers and demand by patients for community-based ART delivery models in case-study districts in Uganda.

## Data Availability

The datasets generated during and/or analyzed during the current study are not publicly available due to ethical reasons but are available from the corresponding author on reasonable request.

## Abbreviations

AIDS: Acquired Immune Deficiency Syndrome
ART: Anti-retroviral therapy
ARVs: Anti-retrovirals
CCLAD: Community Client-Led ART Delivery
CDDP: Community Drug Distribution Points
DSD: Differentiated Service Delivery
FBIM: Facility Based Individual Management
FBG: Facility Based Group
FTDR: Fast-Track Drug Refill
MOH: Ministry of Health
PEPFAR: The Presidents’ Emergency Plan for AIDS Relief
RA: Research Assistant
SSA: Sub-Saharan Africa
WHO: World Health Organization
